# Changes in Toddler Diet Quality Between Ages 12 and 24 Months Using the Healthy Eating Index-Toddlers-2020: A Secondary Analysis of a Prospective Cohort

**DOI:** 10.1016/j.jand.2026.156407

**Published:** 2026-06-24

**Authors:** Sara A. Fortin-Miller, Amy Herman, Sarah A. Crawford, Alexandra R. Brown, Holly R. Hull

**Affiliations:** Department of Dietetics and Nutrition, University of Kansas Medical Center, Kansas City.; Department of Dietetics and Nutrition, University of Kansas Medical Center, Kansas City.; Department of Obstetrics and Gynecology, University of Kansas Medical Center, Kansas City.; Department of Biostatistics and Data Science, University of Kansas Medical Center, Kansas City.; Department of Obstetrics and Gynecology, University of Kansas Medical Center, Kansas City.

**Keywords:** Toddlers, Diet quality, HEI-2020, Should be HEI-Toddlers-2020, Healthy Eating Index

## Abstract

**Background:**

Taste preferences and eating habits develop during the first 2 years of life and often persist over time, influencing the risk of obesity, cardiovascular disease, and other chronic conditions. Although diet quality is known to decline from early childhood to adolescence, less is known about how dietary patterns evolve during toddlerhood.

**Objective:**

The aim of this study was to assess toddler diet quality using the Healthy Eating Index-Toddlers-2020 (HEI-Toddlers-2020) and characterize longitudinal shifts in dietary components and patterns between ages 12 and 24 months.

**Design:**

This was a secondary analysis of children in a prospective cohort. Dietary recalls were collected at ages 12 and 24 months. HEI-Toddlers-2020 scores quantified diet quality, and dietary patterns were derived using principal component analysis (PCA).

**Participants/setting:**

Of 254 children enrolled in the Growth and Adiposity in Newborns Study cohort in Kansas City, KS, 88 were excluded due to incomplete and/or invalid dietary data, yielding a final analytic sample of 166 children. Data were collected from November 2017 to August 2022.

**Main outcome measures:**

Diet quality was assessed using the HEI-Toddlers-2020 total and component scores. Emerging dietary patterns and shifts over time were identified.

**Statistical analyses performed:**

Paired *t* tests and Wilcoxon signed-rank tests assessed changes in HEI scores with effect sizes expressed as the correlation coefficient *r*. Radar plots visually depicted diet quality changes. PCA with varimax rotation identified dietary patterns, and paired *t* tests evaluated changes in pattern adherence over time.

**Results:**

Mean HEI-Toddlers-2020 scores decreased from 59.8 at age 12 months to 57.1 at age 24 months (*P* = .03; *d* = .17), but the difference was not significant after false discovery rate adjustment. Total HEI scores were below recommended levels at both time points. Component-level changes included decreased intake of total vegetables (*P* < .001; *r* = 0.33), and increased intake of refined grains (*P* =.01; *r* = 0.21) and added sugars (*P* < .001; *r* = 0.62). PCA at 12 months identified 4 dietary patterns: PC1 = high unsaturated fat, low dairy/saturated fat; PC2 = vegetable and plant protein; PC3 = fruit-rich, low added sugars; and PC4 = low whole grain, high refined grain, and sodium. From ages 12 to 24 months, mean adherence scores decreased for PC1 (mean difference = −0.66; *P* < .001), PC2 (mean difference = −0.40; *P* = .02), and PC3 (mean difference = −0.61; *P* < .001), and increased for PC4 (mean difference = 0.38; *P* = .002).

**Conclusions:**

Toddler diet quality was below recommendations at both ages 12 and 24 months. Modest changes over time reflected a consistent directional shift in dietary components and patterns, including reduced vegetable intake and increased consumption of refined grains and added sugars, suggesting early emergence of less healthful dietary patterns rather than a large decline in overall diet quality. Findings underscore the importance of early dietary interventions to support healthy eating trajectories.

UNHEALTHFUL DIETARY PATTERNS AND LOW DIET quality are prevalent among US children aged 2 years and older.^1,2^ Current dietary intakes indicate poor alignment with the Dietary Guidelines for Americans (DGAs) from an early age.^3^ The average Healthy Eating Index (HEI) score for children aged 2 to 4 years is 61 out of 100, reflecting suboptimal adherence to recommended dietary patterns.^2^ Few studies have focused on the diet quality of children younger than age 2 years, despite being a critical developmental period during which eating behaviors and food preferences are first established and can persist over time.^4–6^ Furthermore, diet quality tracks and declines from early childhood into adolescence.^2,7^ Poor diet quality in early life is associated with an increased risk of obesity, cardiovascular disease, and other chronic conditions later in life.^8–11^ These findings highlight toddlerhood as a sensitive window during which early dietary transitions may shape long-term nutritional trajectories. Understanding dietary patterns and diet quality during toddlerhood is therefore important for informing interventions aimed at promoting healthier trajectories and reducing long-term disease risk.

The recent release of the HEI-Toddlers-2020 offers a standardized tool for assessing toddler diet quality that is developmentally appropriate and aligned with the DGAs, the primary federal source of guidance on healthful eating patterns across the lifespan.^2,12^ Studies to date have cross-sectionally described diet quality using the HEI-Toddlers-2020.^13–17^ No studies have examined longitudinal changes in diet quality with a single cohort across toddlerhood. Toddlerhood represents a window of opportunity to promote positive nutritional trajectories that benefit longitudinal quality of life.^18,19^ The transition from infancy to toddlerhood involves rapid shifts in feeding practices, including weaning, exposure to family foods, childcare feeding environments, and increasing access to discretionary foods.^20–22^ These transitions may represent the mechanism through which nutritionally suboptimal dietary patterns begin to emerge, rather than random fluctuation in intake.^23,24^ Therefore, it is important to identify and describe early dietary patterns and shifts in components of diet quality to help inform interventions aimed at improving children’s eating behaviors and consequently later health outcomes.^25^

To address these gaps in the literature, this descriptive study longitudinally evaluated toddler diet quality at ages 12 and 24 months using the HEI-Toddlers-2020. Principal component analysis (PCA) of HEI-Toddlers-2020 component scores was then used to identify emerging dietary patterns and to characterize changes in dietary structure over time, rather than relying solely on total HEI score change.

## METHODS

### Study and Participants

This secondary analysis assessed offspring from the Growth and Adiposity in Newborns Study (GAINS) (R01DK118220),^26^ a longitudinal follow-up to a docosahexaenoic acid (DHA) supplementation randomized controlled trial—the Assessment of Docosahexaenoic Acid on Reducing Early Preterm Birth (ADORE) (R01HD83292).^27^ The primary outcomes of both ADORE and GAINS have been previously published.^28,29^ Briefly, the ADORE trial was a multisite, double-blind, controlled, Phase 3 trial in which pregnant persons were randomized to either receive a high (1000 mg/day) or low (200 mg/day) dose of DHA. Those who were aged 18 years or older, between 12 to 20 weeks pregnant, available by telephone, English- or Spanish-speaking, and agreed to consume study capsules were eligible to enroll. Anyone pregnant with multiple fetuses, unwilling to discontinue the use of another supplement with DHA, or who had an allergy to any component of the DHA product was excluded. Only the participants enrolled in ADORE at the Kansas City, KS, site were invited to enroll in GAINS. GAINS study visits occurred between November 2017 and August 2022. The ADORE and GAINS trials are registered at ClinicalTrials.gov as NCT02626299 and NCT03310983, respectively. The ADORE and GAINS trials were approved by the University of Kansas Medical Center Institutional Review Board. Before any data collection, all participants provided written informed consent for ADORE, and both parents provided written informed consent for their offspring enrolled in GAINS.

**Figure 1.**
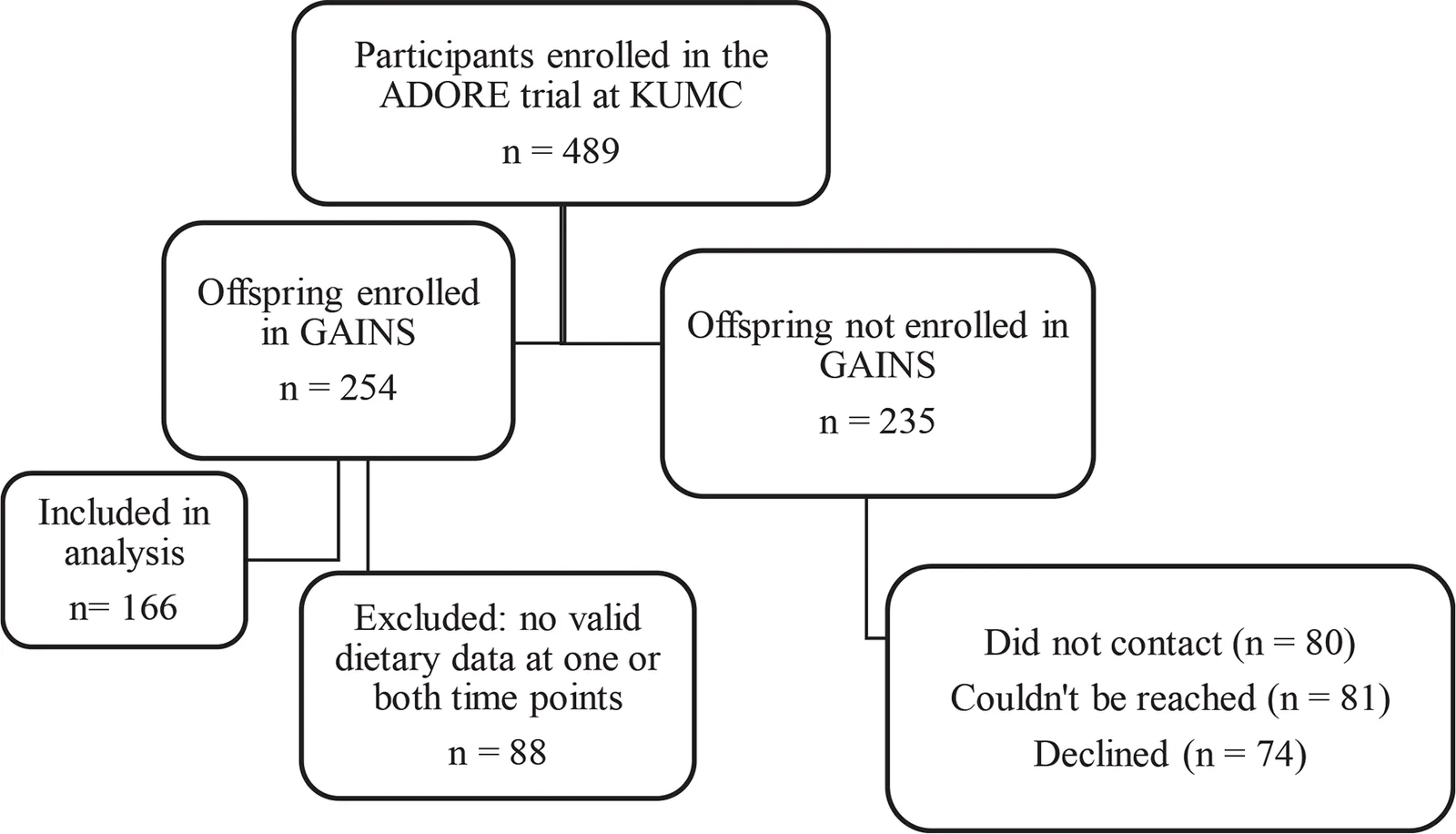
Consolidated Standards of Reporting Trials flow diagram depicting participant enrollment in the Growth and Adiposity in Newborns Study (GAINS) and inclusion in the final analytic sample for this secondary analysis of toddler diet quality at ages 12 and 24 months. Of the 489 participants enrolled in the Assessment of Docosahexaenoic Acid on Reducing Early Preterm Birth (ADORE) trial at the University of Kansas Medical Center (KUMC), Kansas City, KS, site, 254 participants enrolled their offspring in GAINS. The final analytic sample included 166 toddlers with complete and valid 24-hour dietary recall data at both time points; 88 participants were excluded due to missing or invalid dietary intake data at 1 or both assessments. This cohort was used to evaluate longitudinal changes in diet quality between ages 12 and 24 months using the Healthy Eating Index-Toddlers-2020.

See Figure 1 for the Consolidated Standards of Reporting of Trials framework of the sample. Of 489 participants who enrolled in ADORE at the Kansas City site, 254 enrolled their offspring in GAINS, and 233 of the GAINS enrollees had complete and valid dietary intake data at ages 12 months or 24 months. The final analytic sample (n = 166) included those with complete and valid dietary intake data at both time points. Demographic information, including race and ethnicity, language, annual household income, and education of the gestational parent were self-reported. Infant characteristics, including gestational age at birth, birth weight, and birth length, were collected from medical records.

### Dietary Assessment

Dietary intake was assessed by the collection of 1 multiple-pass 24-hour dietary recall by trained personnel at child age 12 months and 24 months. Assessment of dietary intake using 24-hour dietary recalls is a widely used method to estimate nutrient intake in children ages 0 to 24 months.^30–33^ Caregivers were asked to report all foods and beverages the child consumed in the previous day, including those consumed outside the home (eg, at daycare). When applicable, caregivers relied on information provided by daycare staff to complete the recall. To reflect the marketplace throughout the study, dietary intake data were analyzed using Nutrition Data System for Research (NDSR) software versions 2018–2021, developed by the Nutrition Coordinating Center, University of Minnesota, Minneapolis, MN. Final calculations were completed using NDSR version 2021.^34–37^

Diet quality was assessed using NDSR output files and the HEI-Toddlers-2020 scoring standards.^12^ Total and individual component scores were calculated using the simple HEI scoring algorithm,^38^ which applies a density-based approach to account for energy intake. Modeled after the HEI-2020,^39^ the HEI-Toddlers-2020 includes the same 13 components—9 adequacy components (total fruits, whole fruits, total vegetables, greens and beans, whole grains, dairy, total protein foods, seafood and plant proteins, and fatty acids) and 4 moderation components (refined grains, sodium, added sugars, and saturated fats). This standardized tool excludes intake from human milk or infant formula to focus on complementary food consumption and has demonstrated validity and reliability.^14^ Scores range from 0 to 100, with higher scores indicating better overall diet quality.^12,39–41^

### Statistical Analyses

Means ± SD or frequencies were computed for participant characteristics. To assess potential bias from missing dietary data, baseline characteristics were compared between participants in the final analytic sample (n = 166) and those excluded due to incomplete dietary data (n = 88) from the GAINS cohort using independent samples *t* tests and χ^2^ tests. In the final analytic sample, changes in HEI-Toddlers-2020 total scores from ages 12 to 24 months were evaluated with paired samples *t* tests. Due to nonnormal distributions, median component scores were compared using Wilcoxon signed-rank tests. Cohen’s *d* was used to estimate the effect size for the total score (paired *t* test). For component scores (Wilcoxon signed-rank tests), effect sizes were expressed as the correlation coefficient *r*. Radar plots were generated to depict diet quality patterns using the HEI-Toddlers-2020 components. To compare the data between children ages 12 and 24 months, each component score was expressed as a percentage of its maximum possible value and plotted along its respective axis. Statistical significance was set at *P* < .05, with Benjamini-Hochberg false discovery rate (FDR) correction applied for multiple comparisons where appropriate.

PCA with varimax rotation was conducted using the 12-month HEI-Toddlers-2020 component scores to identify baseline dietary patterns based on the components with the greatest variability in intake. This approach enabled examination of co-occurring dietary behaviors and overall dietary pattern structure, rather than focusing solely on changes in individual component scores. All 13 HEI-Toddlers-2020 components at baseline were included in a single PCA. To aid interpretation and place all components on a consistent intake-oriented scale, the 4 moderation components (refined grains, sodium, added sugars, and saturated fat) were multiplied by −1 so that higher values indicated higher intake across all components. This ensured that all adequacy and moderation components could be interpreted consistently: positive loadings reflected higher intake, and negative loadings reflected lower intake. The number of dietary patterns was identified based on factors with initial eigenvalues >1.0 and breaks in the scree plot. Patterns were described in relation to the HEI components that loaded more positively or negatively for the respective patterns. Component loadings with an absolute value ≥0.4 are considered meaningful contributors to a factor and were used to define and describe each dietary pattern.^42^ Components with smaller loadings contribute less and are generally not reported to avoid overinterpretation of weak associations. To enable within-person longitudinal comparisons, 24-month HEI-Toddlers-2020 component scores were standardized using the 12-month means and SD to generate *z* scores. The 12-month PCA loadings were then applied to these standardized 24-month values to calculate comparable 24-month pattern scores. This fixed-loading approach allowed longitudinal differences in pattern scores to be interpreted as changes in adherence to the same baseline-defined dietary patterns over time, rather than as changes in factor structure arising from separate PCAs at each time point. Details of the standardization procedure and the resulting projected 24-month component distributions are provided in Table 1 (available at www.jandonline.org). Paired *t* tests assessed changes in adherence to each dietary pattern between ages 12 and 24 months.

To assess whether dietary pattern structure differed by age, PCA was also conducted separately at the 12- and 24-month time points (Table 2, available at www.jandonline.org). At both time points, 4 factors were retained based on initial eigenvalues >1.0 and visual inspection of the scree plots. These time point-specific PCAs were used to provide empirical context regarding continuity in dietary structure across time points but were not used for the primary longitudinal comparisons. The primary longitudinal analyses were based on the projection of standardized 24-month component scores onto the 12-month PCA loadings described above.

## RESULTS

The sample included a similar proportion of male and female children (46.4% male, 53.6% female). Nearly half of households reported an annual income below $50 000 (45.2%). Most gestational parents had more than a high school education (72.3%), and more than half held a bachelor’s degree or higher (54.8%). The sample was predominantly White (67.5%) but ethnically diverse, with 38.6% identifying as Hispanic or Latino and 23.5% primarily Spanish-speaking. To assess generalizability, the final analytic sample (n = 166) was compared with excluded GAINS participants (excluded for incomplete dietary data) and national US Census data.^43–46^ Baseline differences between included and excluded participants were significant for infant sex, household income, and the gestational parent’s education (Table 3). Compared with Census data, the sample had a similar distribution of infant sex but differed significantly in annual household income, gestational parent’s education, ethnicity, race, and language (Table 4, available at www.jandonline.org).

The HEI-Toddlers-2020 total and component scores are presented in Table 5. The total score decreased from 59.8 to 57.1 (*t*[165] = 2.2; *P* = .03; *d* = 0.17). Although the mean difference reached statistical significance, the effect size was small, and the difference was not significant after FDR adjustment, indicating modest changes in overall diet quality. As a reminder for the interpretation of the HEI, a higher total score indicates better adherence to the DGAs. For the adequacy components, a higher score indicates higher intake or better adherence to the DGAs. For the moderation components, because they represent foods to limit for good health (eg, added sugars), a higher score indicates lower intake or better adherence to the DGAs. After applying the Benjamini-Hochberg FDR correction for multiple comparisons, decreases in component scores were observed for total vegetables (*z* = −4.24; *P* < .001; *r* = 0.33), refined grains (*z* = −2.71; *P* = .01; *r* = 0.21), and added sugars (*z* = −8.02; *P* < .001; *r* = 0.62). In contrast, better adherence to the DGAs was found for dairy, with an increase in the component score from ages 12 to 24 months (*z* = −6.01; *P* < .001; *r* = .47).

**Figure 2.**
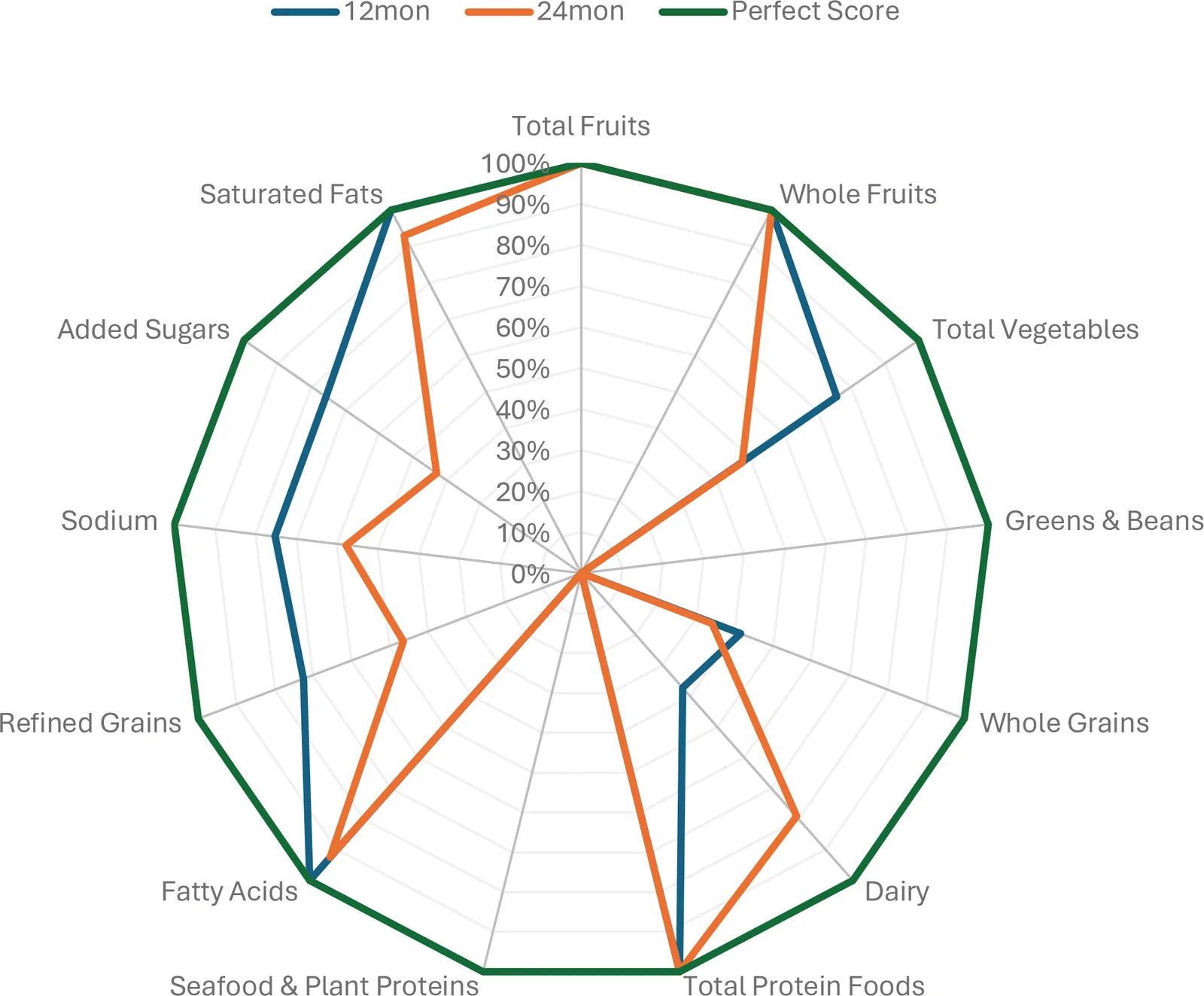
Radar plot of the Healthy Eating Index-Toddlers-2020 (HEI-Toddlers-2020)^a^ median component scores expressed as a percentage of their maximum value from the Growth and Adiposity in Newborns Study (GAINS) participants at ages 12 and 24 months. ^a^Higher total scores indicate better adherence to the Dietary Guidelines for Americans. For adequacy components, higher scores reflect greater intake; for moderation components (eg, added sugars, sodium, refined grains, and saturated fats), higher scores reflect lower intake.

Radar plots illustrating diet quality patterns are shown in Figure 2. The green line around the perimeter represents a perfect score for each component. From age 12 to 24 months, scores for added sugars, refined grains, and total vegetables decreased, whereas the score for dairy increased. In other words, consumption of added sugars, refined grains, and dairy increased, whereas vegetable intake decreased. The remaining components showed minimal variation between time points, with all exhibiting a difference of ≤8% in their median scores.

### Dietary Patterns

PCA with varimax rotation identified 4 distinct dietary patterns at 12 months, together explaining 66.5% of the total variance in HEI-Toddlers-2020 component scores (Table 6). The Kaiser-Meyer-Olkin measure of sampling adequacy was 0.63, indicating a meritorious level of sampling adequacy. Bartlett’s test of sphericity was significant (*P* < .001), suggesting the correlation matrix was suitable for factor analysis. As a reminder, moderation components (refined grains, sodium, added sugars, and saturated fats) were inverted so that positive loadings indicated higher intake and negative loadings indicated lower intake across all components. The first pattern (PC1) was characterized by high fatty acids (0.89) and low dairy (−0.88) and saturated fats (−0.87), representing a high unsaturated fat, low dairy/saturated fat pattern. The second pattern (PC2) reflected a vegetable- and plant-protein-rich pattern, with high loadings for greens and beans (0.85), seafood and plant protein (0.81), total protein foods (0.65), and total vegetables (0.50). The third pattern (PC3) was fruit-rich and low in added sugars, with high loadings for total fruits (0.91) and whole fruits (0.89) and a negative loading for added sugars (−0.47). The fourth pattern (PC4) indicated low whole-grain intake and high refined-grain and sodium intake, with a negative loading for whole grains (−0.77) and positive loadings for sodium (0.75) and refined grains (0.61).

**Figure 3.**
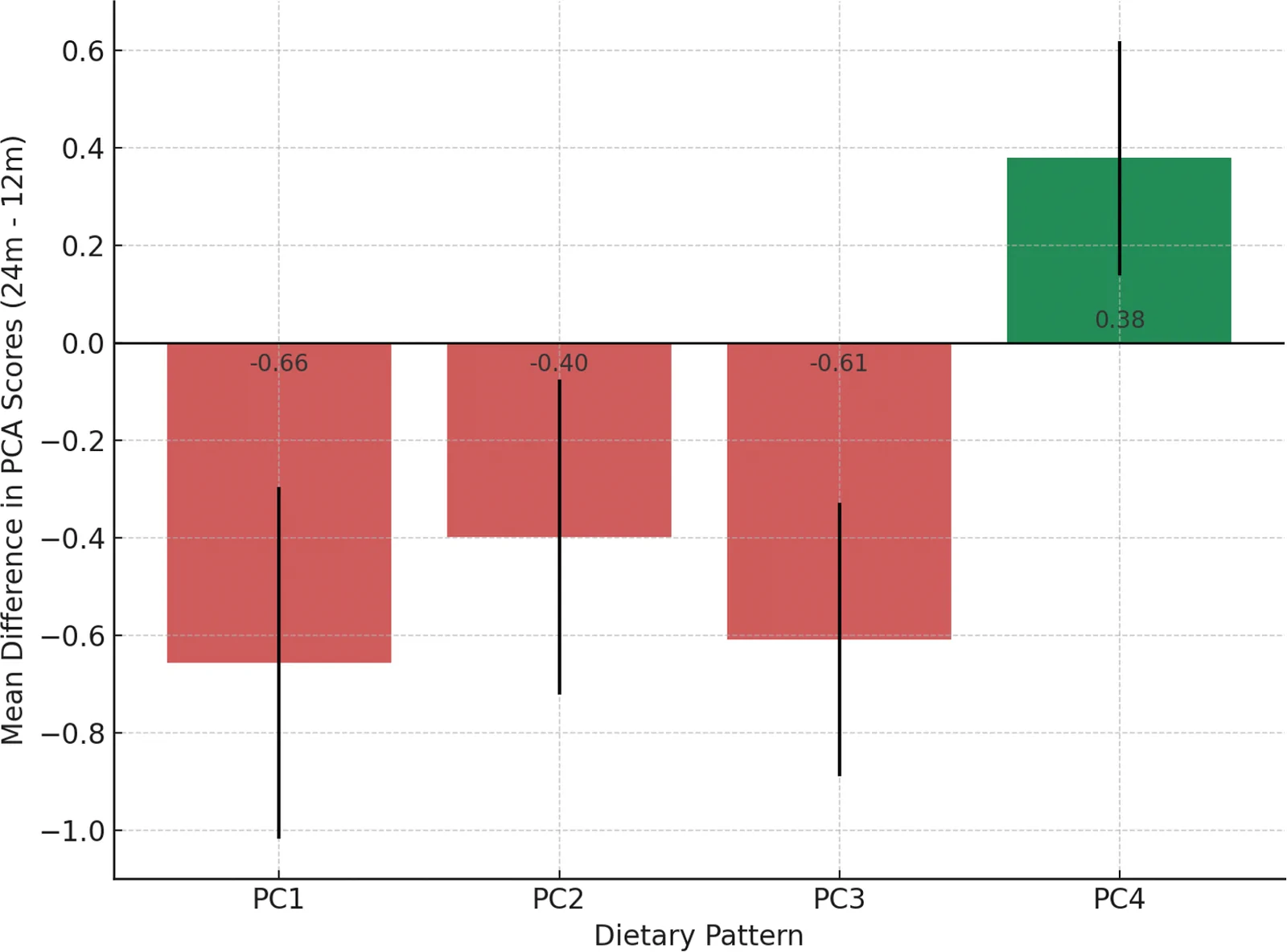
Mean changes in principal components (PC) analysis (PCA)-derived dietary pattern scores between ages 12 and 24 months in toddlers from the Growth and Adiposity in Newborns Study (GAINS).^a a^Factor scores declined for PC1 (*P* < .001), PC2 (*P* = .02), and PC3 (*P* < .001), indicating shifts toward poorer fat quality; reduced intake of vegetables, seafood, and plant proteins, and total protein; and lower fruit intake with higher added sugars, respectively. In contrast, PC4 scores increased (*P* = .002), reflecting greater adherence to a pattern high in refined grains and sodium and low in whole grains.

Between ages 12 and 24 months, significant shifts in dietary patterns were observed (Figure 3). Factor scores declined for PC1 (mean difference = −0.66; *P* < .001), PC2 (mean difference = −0.40; *P* = .02), and PC3 (mean difference = −0.61; *P* < .001), indicating lower fat quality (low unsaturated fats and/or high dairy and saturated fats); reduced intake of vegetables (including greens and beans), seafood and plant proteins, and total protein foods; and low fruit intake with high added sugars, respectively. In contrast, PC4 scores increased (mean difference = 0.38; *P* = .002), reflecting greater adherence to a pattern high in refined grains and sodium and low in whole grains.

In separate exploratory PCAs conducted at each time point, both the 12-month and 24-month PCAs retained 4 factors based on initial eigenvalues >1.0 and visual inspection of the scree plots (Table 2, available at www.jandonline.org). The separate PCAs identified overlapping dietary domains across time points, including fruit, vegetable and plant protein, and fat-related patterns. Some component loadings were redistributed at 24 months, such that the fat-related pattern broadened to include discretionary components and the grain/sodium-related pattern shifted toward a protein/sodium-related pattern.

## DISCUSSION

This is the first known study to use the recently developed HEI-Toddlers-2020^12^ to longitudinally assess changes in toddler diet quality within the same cohort. We examined a critical developmental period when most children transition to table foods by age 12 months^20^ and establish foundational eating habits by age 24 months.^2,47^ Overall diet quality was low at both time points, and the observed change in total HEI scores from ages 12 to 24 months was modest, declining from 59.8 to 57.1; however, this difference was not significant after FDR adjustment. This small shift reflects variation within already low-quality dietary patterns rather than a clinically meaningful deterioration in overall diet quality. However, component-level analyses revealed a consistent directional pattern, with lower vegetable intake and higher intakes of refined grains and added sugars over time. These shifts mirror dietary patterns associated with poor diet quality in older children and adolescents, including lower intake of nutrient-dense foods and higher intake of discretionary foods, and suggest that nutritionally suboptimal dietary patterns may begin to emerge during this transitional developmental period.^1,48^ Although some dietary change is expected as children move from infant feeding to family foods, the observed movement toward discretionary foods suggests that early dietary transition may preferentially favor lower-quality substitutions rather than nutritionally neutral exchanges. Although the HEI-Toddlers-2020 does not assign letter grades, application of the National Institutes of Health “A-F” grading scale used for individuals older than age 2 years would classify both scores as an “F,” reflecting very poor diet quality^49^ and emphasizing the need for improvement in dietary patterns during early life.

Dietary patterns at 12 months were characterized by high intake of adequacy components (unsaturated fats, vegetables, total protein foods, and fruits) alongside lower intake of moderation components (saturated fat and added sugars). Using the baseline-defined PCA structure, adherence decreased from 12 to 24 months for patterns characterized by unsaturated fats, vegetables and plant proteins, and fruits, whereas adherence increased for the pattern characterized by higher refined grains and sodium and lower whole grains. These PCA findings reflect shifts in adherence to multicomponent dietary patterns and should be interpreted separately from the individual component-level analyses, which indicated that the observed pattern shifts were mainly driven by increased dairy intake, decreased vegetable intake, and increased intakes of refined grains and added sugars. Although the increase in dairy intake may partly reflect the transition away from breastmilk or formula, the HEI-Toddlers-2020 does not distinguish between sweetened and unsweetened dairy products. The concurrent increase in added sugars suggests that at least part of this increase may reflect greater intake of sweetened dairy products (eg, flavored milks and sweetened yogurts), representing a shift in food sources rather than a true improvement in diet quality. Overall, findings reveal a transition from adequacy-based patterns toward less favorable moderation-based patterns between 12 and 24 months. These changes likely reflect both developmental transitions (eg, increasing autonomy and broader food exposure) and environmental influences on food availability and selection, rather than a uniform decline in overall diet quality.^22,50^ Such behavioral shifts may outweigh sociodemographic influences, although future studies with larger samples could explore potential moderators of dietary change.

Five prior studies have reported toddler diet quality scores using the newly developed HEI-Toddlers-2020.^13–17^ All were cross-sectional, assessing diet quality in children aged 12 to 23.9 months using data from the 2016 Feeding Infants and Toddlers Study (FITS)^13^ and National Health and Nutrition Examination Survey (NHANES) 2011–2018, 2017–2018, 2017–2020, and 2013–2020.^14–17^ Consequently, these studies could not evaluate changes in diet quality within the same children over time. In contrast, the present study examined longitudinal changes from ages 12 to 24 months. Total HEI-Toddlers-2020 scores declined from 59.8 at age 12 months to 57.1 at age 24 months, although this difference was not significant after FDR adjustment, with low diet quality at both ages. These values were similar to NHANES 2011–2018, 2017–2020, and 2013–2020 scores (62.9, 56.5, and 55.3–59.5, respectively).^14,16,17^ However, they were 10 to 14 points lower than scores reported in the NHANES 2017–2018 and FITS cohorts (67.7 and 71.2, respectively).^13,15^ Consistent with the present findings, Kay and colleagues^13^ reported low adherence in the FITS cohort for total vegetables, whole grains, added sugars, and refined grains. Lerman and colleagues^14^ also found poor adherence for vegetables, seafood and plant proteins, whole grains, and added sugars in NHANES 2011–2018. In the present analysis, adherence was highest for total fruits, whole fruits, and total protein foods when summarized using median component scores (Figure 2). Fatty acids and saturated fats also reached high median scores at 12 months. In contrast, adherence was lower for vegetables, whole grains, dairy (12 months only), refined grains, sodium, and added sugars. By comparison, studies summarizing adherence using mean component scores have also reported higher adherence for selected components; Kay and colleagues^13^ found ≥86% adherence for total fruits, whole fruits, total protein foods, dairy, and seafood and plant proteins in the FITS cohort, whereas Zimmer and colleagues^15^ reported >80% adherence for seafood and plant proteins in NHANES 2017–2018.^15^ Differences in summary metrics, analytic approach, and population characteristics should be considered when interpreting these comparisons. Collectively, these findings indicate that although some toddler dietary components align more closely with the DGAs than others, low diet quality is already established by age 12 months and persists through age 24 months, with emerging shifts toward poorer dietary composition during this interval.

Multiple studies using other diet quality indexes have similarly reported poor diet quality in toddlers. Differences in scoring criteria across indices should be considered when comparing findings across the literature. Meyerkort and colleagues^6^ used the Raine Eating Assessment in Toddler Score index to assess changes in diet quality from ages 1 to 3 years. Consistent with the present results, diet quality declined over time (1-year-old boys: 41.7 out of 70, 3-year-old boys: 36.7 out of 70; 1-year-old girls: 41.7 out of 70, 3-year-old girls: 38.3 out of 70). The decline in diet quality was similarly related to increased consumption of unhealthy snack foods and sugar-sweetened beverages and decreased intake of whole grains and vegetables. Ríos and colleagues^51^ applied the Diet Quality Index Score to toddlers aged 12 to 24 months and reported a mean score of 25.7 out of 55, with 50.4% of participants classified as having “poor” diet quality. Their findings reflected overconsumption of 100% fruit juice, proteins, refined grains, and milk. Kay and colleagues^52^ used the Toddler Diet Quality Index to assess diet quality in toddlers aged 12 to 23.9 months. The average total score was 48.6 out of 100, indicating poor diet quality, with low intake of vegetables, whole grains, and seafood and plant proteins, alongside excessive added sugars and refined grains. Hamner and Moore^53^ used a modified version of the Diet Quality Index Score to assess diet quality cross-sectionally in 1-year-old children (aged 12 to 23 months) and 2- to 3-year-old children (aged 24 to 47 months) using NHANES data. Total scores were higher in the 1-year-old children (23.9 out of 45) compared with the 2- to 3-year-old children (21.4 out of 45). The decline was driven by decreased protein and milk intake and increased intake of sugar-sweetened beverages and salty snacks in the 2- to 3-year-old children. Despite methodological differences across indices and cohorts, this and prior studies consistently demonstrate low diet quality in US toddlers,^14,51–53^ with progressive increases in discretionary foods and reductions in nutrient-dense foods as children age.^53^

At age 12 months, PCA identified 4 distinct dietary patterns. The first was characterized by high fatty acid intake with low dairy and saturated fat (fats pattern). The second included high intake of greens and beans, seafood and plant proteins, total protein foods, and vegetables (vegetables and protein pattern). The third reflected high fruit intake with low added sugars (fruit pattern). The fourth was characterized by high sodium and refined grains with low whole grains (grains pattern). These patterns are consistent with previous studies in infants and toddlers that reported combinations of vegetables, meats, and fruits as dominant patterns.^54–59^ In this cohort, the “greens and beans” component contributed notably to variability, consistent with its high loading in the PCA. This may reflect cultural influences because nearly 40% of participants identified as Hispanic, and beans are a common staple food in Hispanic diets. Other studies have similarly found that participants exhibit dietary patterns native to their culture or region.^60,61^ For example, a Brazilian cohort showed a meat-and-vegetable pattern in infancy that was no longer present by age 48 months.^56^ In the present cohort, the vegetables and protein pattern score similarly declined by age 24 months, although component-level trends showed that greens and beans intake did not significantly decrease. Scores for the fats and fruit patterns also declined. In contrast, the grains score increased, indicating greater adherence to a low whole grain, high refined grain, and sodium pattern. Together, these results suggest a coordinated transition from adequacy-oriented dietary patterns toward less favorable, moderation-based patterns during a critical developmental period, rather than isolated changes in single dietary components.

### Strengths and Limitations

This study had several strengths. Following the same children from 12 to 24 months allowed for longitudinal assessment of diet quality, capturing within-individual changes rather than relying on cross-sectional comparisons. This approach reduced confounding from between-subject variability and provided a clearer understanding of how eating habits evolve during a critical window of dietary and behavioral development. The use of the recently released HEI-Toddlers-2020^12^ enabled a comprehensive evaluation of diet quality that is specifically tailored to the nutritional needs of toddlers. Applying PCA with varimax rotation provided additional insight into the structure of toddlers’ diets by identifying clusters of food groups that tend to be consumed together, allowing us to detect shifts in underlying dietary patterns that may not be evident from total HEI scores alone. The cohort’s ethnic diversity enhanced the applicability of the findings to populations that are frequently underrepresented in pediatric nutrition research, particularly in studies of early dietary development. Furthermore, the study employed rigorous dietary assessment methods, including 24-hour recalls, which reduce recall bias and improve accuracy in estimating usual intake.^62–64^ Overall, these strengths support the interpretation that the observed changes reflect emerging dietary structure rather than measurement artifact, while still acknowledging modest effects sizes in the total HEI score.

A key limitation of this study is the use of a single 24-hour dietary recall at each time point, which may not fully capture usual intake or reflect day-to-day variation. Although 24-hour recalls are widely used and generally yield more accurate estimates with less reporting bias than diet records,^62–64^ collection of at least 2 recalls is recommended for estimating usual energy and nutrient intake.^65^ Relying on a single recall may reduce the precision of HEI scores and weaken the stability of derived dietary patterns by increasing the influence of within-person variability. Statistical methods such as the National Cancer Institute^66^ or Iowa State University^67^ approaches can adjust for this variability at the group level, but they require multiple days of intake data and were not feasible in this study. Dietary data collection in this age group also poses practical challenges, particularly when meals are consumed outside the home. Many toddlers attend daycare, and caregivers may have limited information on what and how much their child consumed, introducing potential measurement error. Although parental dietary recalls show mixed validity across studies,^68–70^ a review by Foster and Bradley^71^ emphasized their continued value, noting that no superior alternative currently exists. These sources of measurement variability may partially contribute to modest changes in total HEI score but are unlikely to fully explain the consistent direction of component-level shifts observed across multiple dietary domains. Another limitation is that this was a secondary analysis, and the parent study was not specifically designed to examine changes in toddler diet quality. As a result, some aspects of data collection, such as the timing of dietary assessments and reliance on a single recall per time point, may limit sensitivity to detect precise trends. Nonetheless, the availability of prospectively collected dietary data at 2 developmentally important ages (12 and 24 months) offers valuable insight into early diet transitions. Lastly, PCA-derived dietary patterns are inherently data-driven and may vary depending on analytic decisions (eg, number of components retained, rotation method, and loading thresholds) that may influence reproducibility. The fixed-loading approach used for within-person longitudinal comparison also assumes partial stability of dietary structure over time; accordingly, longitudinal differences should be interpreted as shifts in adherence to baseline-defined dietary constructs (and their dietary composition) rather than strict invariance of pattern structure during this period of rapid dietary diversification.

## CONCLUSIONS

Using the recently released HEI-Toddlers-2020, a standardized tool to assess toddler diet quality and adherence to the DGAs, this study found that toddler diet quality was poor at both ages 12 and 24 months in a cohort of 166 toddlers in Kansas City, KS. Although changes in total HEI scores were modest, the consistent direction of change across multiple dietary components suggests an early shift toward less healthful dietary patterns during toddlerhood. Notable longitudinal changes from ages 12 to 24 months included decreased vegetable intake and increased intakes of refined grains and added sugars. Rather than indicating a large decline in overall diet quality, these findings point to the early emergence of nutritional suboptimal patterns within an already low-quality dietary context. Together, the findings highlight specific dietary components that may be targeted in future interventions aimed at improving early childhood nutrition during this critical developmental period.

## Supplementary Material

1

Tables 1, 2, and 4 are available at www.jandonline.org

## Figures and Tables

**Table 3.** Sociodemographic characteristics of participants enrolled in the Growth and Adiposity in Newborns Study (GAINS) and those with valid dietary data at 12 and 24 months

	Final analytical sample (N = 166)^a^	Excluded sample (n = 88)^b^	*P* value^c^

		*mean ± SD*	
Gestational age at birth (wk)	38.7 ± 1.7	38.7 ± 1.3	.98
Birth weight (g)	3274.5 ± 532.0	3406.6 ± 507.2	.05
Birth length (cm)	50.0 ± 2.9	50.6 ± 2.5	.11
		*n (%)*	
**Infant sex**			
Male	77 (46.4)	54 (61.4)	.03
Female	89 (53.6)	34 (38.6)	
**Annual household income**			
<$50 000	75 (45.2)	53 (60.2)	.03
≥$50 000	91 (54.8)	35 (39.8)	
**Education of gestational parent**			
High school or General Educational Development	46 (27.7)	42 (47.7)	.002
Tech or some college	29 (17.5)	15 (17.0)	
Bachelor’s degree	51 (30.7)	24 (27.3)	
Beyond bachelor’s degree	40 (24.1)	7 (8.0)	
**Ethnicity**			
Not Hispanic or Latino	102 (61.4)	45 (51.1)	.14
Hispanic or Latino	64 (38.6)	42 (47.7)	
Unknown/not reported	0 (0)	1 (1.1)	
**Race**			
American Indian/Alaskan Native	5 (3.0)	2 (2.3)	.34^d^
Asian	2 (1.2)	1 (1.1)	
Black or African American	13 (7.8)	10 (11.4)	
White	112 (67.5)	54 (61.4)	
More than one race	26 (15.7)	9 (10.2)	
Unknown/not reported	8 (4.8)	12 (13.6)	
**Language**			
English	127 (76.5)	62 (70.5)	.30
Spanish	39 (23.5)	26 (29.5)	

a Offspring enrolled in the GAINS study who had valid dietary data at both 12 and 24 months.

b Offspring enrolled in the GAINS study who did not have valid dietary data at both time points and were therefore excluded from analysis.

c The total GAINS cohort included 254 offspring; *P* values reflect comparisons between participants in the analytic sample (N = 166) and those excluded from the GAINS cohort (n = 88). Independent samples *t* tests were used for continuous variables and χ^2^ tests for categorical variables.

d Collapsed into “White” and “Other” to meet χ^2^ test assumptions (all expected counts >5).

**Table 5.** Healthy Eating Index-Toddlers-2020 Component and Total Scores at ages 12 and 24 months (N = 166) in the Growth and Adiposity in Newborns Study

	12 mo	24 mo	*P* value^a^	Effect size^b^

	*mean ± SD*			
Total score^c^	59.8 ± 14.2	57.1 ± 13.5	.03	0.17
	*median (IQR)*			
**Adequacy components** ^d^				
Total fruits	5.0 (2.4)	5.0 (2.3)	.65	0.04
Whole fruits	5.0 (2.7)	5.0 (1.8)	.50	0.05
Total vegetables	3.8 (3.7)	2.4 (2.7)	**< .001** ^e^	0.33
Greens and beans	0.0 (5.0)	0.0 (5.0)	.86	0.01
Whole grains	4.2 (9.8)	3.4 (6.6)	.05	0.16
Dairy	3.7 (8.6)	7.9 (5.7)	**< .001** ^e^	0.47
Total protein foods	5.0 (2.8)	5.0 (2.2)	.40	0.07
Seafood and plant proteins	0.0 (5.0)	0.0 (5.0)	.38	0.07
Fatty acids	10.0 (7.3)	9.2 (6.9)	.89	0.01
**Moderation components** ^f^				
Refined grains	7.3 (9.6)	4.7 (9.2)	**.01** ^e^	0.21
Sodium	7.5 (8.5)	5.8 (8.7)	.12	0.12
Added sugars	7.6 (3.9)	4.3 (5.0)	**< .001** ^e^	0.62
Saturated fats	10.0 (3.8)	9.3 (4.8)	.43	0.06

a Paired sample *t* tests were used to compare mean total scores between 12 and 24 months, whereas Wilcoxon signed-rank tests were used to compare median component scores.

b Cohen’s *d* was used to estimate the effect size for the total score (paired *t* test). For component scores (Wilcoxon signed-rank tests), effect sizes were expressed as the correlation coefficient *r*.

c Total scores range = 0 to 100.

d Adequacy component scores range = 0 to 5 (0 to 10 for whole grains, dairy, and fatty acids); higher scores indicate higher intake or better adherence to the 2020–2025 Dietary Guidelines for Americans.

e *P* values for component comparisons were adjusted for multiple testing across 14 components using the Benjamini-Hochberg false discovery rate (FDR). Components that remained statistically significant after FDR correction are in boldface type.

f Moderation component scores range = 0 to 10; higher scores indicate lower intake of dietary components to limit or better adherence to the 2020–2025 Dietary Guidelines for Americans.

**Table 6.** Dietary component loadings for the principal components (PC) analysis (PCA) dietary patterns at 12 months in the Growth and Adiposity in Newborns Study^a^

	12 mo (N = 166)^b^			
	Factor 1 (PC1)	Factor 2 (PC2)	Factor 3 (PC3)	Factor 4 (PC4)

Eigenvalue	2.84	2.38	1.91	1.51
% Variance explained	21.9	18.3	14.7	11.6
Fatty acids	**0.89**	0.12	−0.01	0.08
Dairy	**−0.88**	−0.05	−0.07	−0.04
Saturated fat	**−0.87**	−0.03	−0.10	0.01
Greens and beans	0.03	**0.85**	0.102	−0.08
Seafood and plant proteins	0.11	**0.81**	−0.04	−0.12
Total protein foods	0.01	**0.65**	−0.02	0.23
Total vegetables	0.09	**0.50** ^c^	**0.43**	0.12
Total fruits	0.09	−0.06	**0.91**	−0.03
Whole fruits	0.01	−0.06	**0.89**	−0.07
Added sugars	−0.04	−0.25	**−0.47**	0.05
Whole grains	0.22	−0.04	−0.26	**−0.77**
Sodium	0.17	0.19	−0.21	**0.75**
Refined grains	0.30	−0.32	−0.26	**0.61**

a To aid in interpretation of the PCA component loadings, the inverse of the Healthy Eating Index Toddlers-2020 moderation components (refined grains, sodium, added sugars, and saturated fats) was calculated. Therefore, for all components, a positive loading indicates a higher intake, and a lower loading indicates lower intake.

b Values in boldface type represent absolute loadings ≥0.40, indicating that the dietary component made a strong contribution to the respective factor.

c When a component loaded ≥0.40 on multiple factors, it was assigned to the factor with the highest absolute loading.

## RESEARCH SNAPSHOT

### Research Question:

How do toddler diet quality and dietary patterns change between ages 12 and 24 months?

### Key Findings:

In a prospective cohort of toddlers (n = 166), overall diet quality, assessed using the Healthy Eating Index-Toddlers-2020, declined slightly from 59.8 at 12 months to 57.1 at 24 months. However, this change was not statistically significant after false discovery rate adjustment. Significant component-level changes included decreased vegetable intake and increased consumption of refined grains and added sugars. Dietary patterns shifted toward lower fat quality, reduced intake of adequacy components, and increased intake of moderation components.
