# Supplementary material for: Changes in Toddler Diet Quality Between Ages 12 and 24 Months Using the Healthy Eating Index-Toddlers-2020: A Secondary Analysis of a Prospective Cohort
Source: J Acad Nutr Diet. Author manuscript; Available in PMC 2026 Sep 29. (PMC13617658; doi:10.1016/j.jand.2026.156407)
Supplement: Supplementary file 1 [file NIHMS2209576-supplement-1.pdf]

Table 1. Standardization and projection of 24-month Healthy Eating Index-Toddlers-2020 (HEI-Toddlers-2020) component scores using 12-mo principal component (PC) analysis (PCA) loadings in the Growth and Adiposity in Newborns Study (N = 166)

HEI-Toddlers-2020 Component	12 mo	Factor	12-mo PCA loading[a]	24-mo standardized component (*z*)
	Mean ± SD			Mean ± SD[b]
Fatty acids	6.80 ± 4.06	PC1	0.89	−0.01 ± 0.94
Dairy	4.22 ± 4.07		−0.88	0.67 ± 0.79
Saturated fats[c]	−7.60 ± 3.64		−0.87	0.06 ± 0.95
Greens and beans	1.72 ± 2.33	PC2	0.85	−0.004 ± 0.98
Seafood and plant proteins	1.80 ± 2.36		0.81	0.09 ± 1.01
Total protein foods	3.68 ± 2.0		0.65	0.09 ± 0.78
Total vegetables	3.17 ± 1.93		0.50	−0.41 ± 0.84
Total fruits	3.65 ± 2.0	PC3	0.91	0.05 ± 0.90
Whole fruits	3.72 ± 2.13		0.89	0.06 ± 0.90
Added sugars[c]	−6.91 ± 2.87		−0.47	0.91 ± 1.08
Whole grains	4.79 ± 4.10	PC4	−0.77	−0.20 ± 0.89
Sodium[c]	−6.01 ± 4.12		0.75	0.14 ± 0.94
Refined grains[c]	−5.76 ± 4.28		0.61	0.25 ± 0.93

[a]PCA was conducted using 12-month HEI-Toddlers-2020 component scores to define baseline dietary patterns. The 12-month loadings were retained for cross-time comparability and used to project 24-month PC score.
[b]At 24 months, each HEI-Toddlers-2020 component was standardized using the corresponding 12-month mean ± SD (column 2). Comparable 24-month PC scores were calculated as linear combinations of standardized 24-month component values multiplied by the corresponding baseline PCA loadings.
[c]Moderation components (saturated fats, added sugars, sodium, and refined grains) were multiplied by −1 before PCA so that higher values reflect higher intake across all components.

Table 2. Dietary component loadings from separate principal component (PC) analyses (PCA) conducted at 12 and 24 months in the Growth and Adiposity in Newborns Study (GAINS) (n = 166)[a]

	Dietary domain[c,d]			
HEI-Toddlers-2020 component[b]	PC1 Fat-related	PC2 Vegetable and plant protein	PC3 Fruit	PC4 Grain/ sodium-related
**12-mo PCA solution**				
Total fruits			0.91	
Whole fruits			0.89	
Total vegetables		0.50[e]	0.43	
Greens and beans		0.85		
Total protein foods		0.65		
Seafood and plant proteins		0.81		
Dairy	−0.88			
Saturated fats, inverse	−0.87			
Fatty acids	0.89			
Sodium, inverse				0.75
Added sugars, inverse			−0.47	
Whole grains				−0.77
Refined grains, inverse				0.61
**Eigenvalue**	2.84	2.38	1.91	1.51
**% variance explained**	21.9	18.3	14.7	11.6
**Cumulative variance explained (%)**	21.9	40.2	54.9	66.5
	**PC1 Fat/ discretionary**	**PC2 Vegetable and plant protein**	**PC3 Fruit**	**PC4 Protein/ sodium-related**
**24-mo PCA solution**				
Total fruits			0.916	
Whole fruits			0.863	
Total vegetables		0.450		
Greens and beans		0.807		
Total protein foods				0.664
Seafood and plant proteins		0.833		
Dairy	−0.738			
Saturated fats, inverse	−0.753			
Fatty acids	0.702[e]			0.414
Sodium, inverse				0.661
Added sugars, inverse	0.643			
Whole grains				−0.527
Refined grains, inverse	0.599			
**Eigenvalue**	2.58	2.44	1.66	1.29

(*continued on next page*)

Table 2. Dietary component loadings from separate principal component (PC) analyses (PCA) conducted at 12 and 24 months in the Growth and Adiposity in Newborns Study (GAINS) (n = 166)[a] (*continued*)

	PC1 Fat/ discretionary	PC2 Vegetable and plant protein	PC3 Fruit	PC4 Protein/ sodium-related
**% variance explained**	19.9	18.7	12.8	9.9
**Cumulative variance explained (%)**	19.9	38.6	51.4	61.3

[a]Separate PCAs were conducted at ages 12 and 24 months using the same analytic approach as described in the Methods. Both PCAs retained 4 factors based on initial eigenvalues >1.0 and visual inspection of the scree plots. Overlapping dietary domains across time points were identified, particularly fruit, vegetable and plant protein, and fat-related domains, although the fat-related pattern broadened to include discretionary components and the grain/sodium-related domain shifted toward a protein/sodium-related pattern at 24 months.
[b]HEI-Toddlers-2020 = Healthy Eating Index-Toddlers 2020. Moderation components (refined grains, sodium, added sugars, and saturated fats) were multiplied by −1 before PCA so that higher values reflected higher intake across all components.
[c]Loadings with an absolute value ≥0.40 were considered meaningful contributors to factor interpretation and are shown in the table.
[d]PC1-PC4 = principal component factors 1-4.
[e]Components that loaded ≥0.40 on more than 1 factor were interpreted according to the factor with the highest absolute loading.

Table 4. Sociodemographic characteristics of the final analytic sample from the Growth and Adiposity in Newborns Study (GAINS) with valid dietary intake data at ages 12 and 24 months compared with US Census estimates

	Final analytical sample (N = 166)[a]	US Census[b]	*P* value[c]
	n (%)	%	
**Infant sex**			
Male	77 (46.4)	49.5	.42
Female	89 (53.6)	50.5	
**Annual household income**			
<$50 000	75 (45.2)	32.3	< .001
≥$50 000	91 (54.8)	67.7	
**Gestational parent's education**			
High school or GED	46 (27.7)	36.1	< .001
Tech or some college	29 (17.5)	27.7	
Bachelor's degree	51 (30.7)	21.8	
Beyond bachelor's degree	40 (24.1)	14.3	
**Ethnicity**			
Not Hispanic or Latino	102 (61.4)	80.6	< .001
Hispanic or Latino	64 (38.6)	19.4	
**Race**			
American Indian/Alaskan Native	5 (3)	1.0	.001
Asian	2 (1.2)	6.0	
Black or African American	13 (7.8)	12.1	
White	112 (67.5)	60.5	
More than 1 race	26 (15.7)	12.8	
Unknown/Not reported/Other	8 (4.8)	7.6	
**Language**			
English	127 (76.5)	77.5	< .001
Spanish	39 (23.5)	13.7	
Other	0 (0)	8.8	

[a]Offspring enrolled in the GAINS study with valid dietary data at both age 12 and 24 months.
[b]US Census data reflect estimates from the total US population, based on the 2023 American Community Survey.[43-46] These estimates are not limited to households with young children.
[c]χ^2 goodness-of-fit test comparing sample distribution to US Census; $P < .05$ was considered significant.
